# Sclerostin decreases in regular swimmers after ice swimming and is associated with meteorin-like protein serum levels

**DOI:** 10.3389/fphys.2023.1210457

**Published:** 2023-06-26

**Authors:** Shuai Mu, Chongjun Huang, Bobo Chen, Pengyu Zhao, Haoran Xu, Weihao Zhang, Huixu Dai

**Affiliations:** ^1^ Department of Orthopedics, Shengjing Hospital of China Medical University, Shenyang, China; ^2^ Department of Clinical Epidemiology, Shengjing Hospital of China Medical University, Shenyang, China

**Keywords:** sclerostin, metrnl, osteokine, adipomyokine, exercise, cold exposure

## Abstract

**Introduction:** Cold and exercise are two important stimuli affecting the secretion of osteokines and adipomyokines, which often occur simultaneously. However, few studies have investigated the changes in osteokines and adipomyokines induced by exercise during severe cold and their corresponding associations. Therefore, this study aimed to investigate the changes in sclerostin and meteorin-like (metrnl) protein before and after cold exercise (ice swimming [IS]) and observe their correlation.

**Methods:** For this, 56 daily ice swimmers’ data were included in this study. Serum sclerostin and metrnl were measured 30 min before IS and 30 min after. The fat mass, visceral fat area, fat-free mass, skeletal muscle mass, lumbar spine, and femoral neck bone mineral density of the ice swimmers were measured.

**Results:** After IS, sclerostin exhibited significant decreases, whereas metrnl showed no significant change. In addition, the baseline level of sclerostin and the decrease in sclerostin were positively correlated with serum metrnl after adjusting for age, gender, and body composition indicators.

**Discussion:** IS caused a significant decrease in sclerostin but did not affect metrnl. Furthermore, the associations between sclerostin and metrnl suggested a correlation between osteokines and adipomyokines; this encourages future exploration of the interconnection between bone, muscle, and fat, which will be beneficial for identifying potential common therapeutic targets for diseases such as osteoporosis, sarcopenia, and obesity.

## 1 Introduction

Bone and muscle are not only anatomically connected but also exchange biochemical signals that are mutually regulated by their respective secretion of osteokines and myokines (Tagliaferri et al., 2015; Lara-Castillo and Johnson, 2020). The concept of the bone–muscle unit depicts the interaction of bones and muscles as an integrated entity during physiological and pathological changes (Tagliaferri et al., 2015). Indeed, osteoporosis and sarcopenia often coexist in the elderly population (Li et al., 2019). Moreover, adipose tissue is closely associated with the skeletal–muscular system (Kirk et al., 2020). In addition to osteoblasts, muscle cells, and adipocytes are derived from the same type of stem cells (Wang et al., 2021). Adipose and muscle tissues co-secrete several identical cytokines known as adipomyokines, including irisin and meteorin-like (metrnl) protein (Boström et al., 2012; Rao et al., 2014; Fang et al., 2023). These adipomyokines contribute directly to bone metabolic homeostasis and the transformation of white adipose tissue into bone-protecting brown adipose tissue (Colaianni and Grano, 2015; Colaianni et al., 2019). Thus, the three tissues—bone, muscle, and adipose—are interrelated, and the cytokines they secrete also communicate with each other (Kirk et al., 2020).

Cold and exercise are two important stimuli that affect the secretion of osteokines and adipomyokines (Boström et al., 2012; Rao et al., 2014). However, most published studies have only examined the effects of a single stimulus (cold or exercise) on osteokines or adipomyokines. For example, researchers have observed changes in sclerostin and irisin in cold conditions, such as whole-body cryostimulation (−110°C) (Dulian et al., 2015; Śliwicka et al., 2020; Straburzynska-Lupa et al., 2021). Meanwhile, the effects of exercise on osteokines and adipomyokines have been more widely investigated (Görgens et al., 2015; Dolan et al., 2020; Domin et al., 2021). However, reports on the effects of “dual stress” (i.e., cold + exercise) on osteokines and adipomyokines are scarce. We previously reported changes in irisin and bone metabolism markers before and after cold exercise (ice swimming [IS]: winter swimming with a water temperature of <5°C [(Knechtle et al., 2021)]) and observed a correlation between their changes during cold exercise and body composition (Mu et al., 2020; Mu et al., 2021). However, no reported evidence has suggested an association between osteokines and adipomyokines during exercise in cold conditions.

Sclerostin, a prominent osteokine secreted mainly by osteocytes, is one of the most significant discoveries in bone metabolism in recent years (Delgado-Calle and Bellido, 2022). Sclerostin is edited by the *SOST* gene and is a well-established antagonist of the classical Wnt/β-catenin signaling pathway—the signaling axis that is essential for bone, adipose, and energy metabolism (Oniszczuk et al., 2022). Metrnl, a newly discovered adipomyokine similar to irisin, was initially discovered through a spliced isoform of PGC-1a (PGC1-a4) transgenic mice (Rao et al., 2014). Both metrnl and irisin are secreted in response to exercise and cold exposure and promote the transformation of white adipose tissue to brown adipose tissue (Boström et al., 2012; Rao et al., 2014). This effect causes an increase in whole-body energy expenditure, thereby preventing obesity and enhancing glucose tolerance (Boström et al., 2012; Rao et al., 2014).

A correlation between sclerostin and irisin has been described previously (Klangjareonchai et al., 2014). However, it has not been determined whether there was an association between sclerostin and metrnl worth exploring. Novel evidence on the correlation between osteokines and adipomyokines may lead to new insights into potential common therapeutic targets for diseases such as osteoporosis, sarcopenia, and obesity. Moreover, cold and exercise are two common stimuli that cause changes in osteokines and adipomyokines, which often occur simultaneously, and both stimuli can independently affect the expression of sclerostin and metrnl (Rao et al., 2014; Straburzynska-Lupa et al., 2021; Oniszczuk et al., 2022). However, it is still unknown whether exercise in a cold environment elicits marked variations in circulating sclerostin and metrnl. Hence, it is interesting to investigate the changes in sclerostin and metrnl and whether they are correlated under the concurrent stimulation of cold and exercise.

Accordingly, this study aimed to investigate the variations in circulating sclerostin and metrnl levels after IS exercise and the correlation between their baseline levels and the changes caused by IS.

## 2 Materials and methods

### 2.1 Participants and study design

In 2019, we initiated the current observational study to investigate changes in osteokines and adipomyokines before and after IS. All the study subjects were daily participants in IS and were aged >40 years. After providing written informed consent, the participants were subjected to the following exclusion criteria: 1) a history of mental illness or inability to comply with the study, 2) a history of cardiovascular diseases, such as heart attack or stroke, 3) a previous diagnosis of osteoporosis or use of anti-osteoporosis medication or hormonal medication, and 4) those with any health risks associated with participation in this study. In total, 87 ice swimmers (90 volunteered to participate; however, three participants who failed to complete the study were excluded) participated in this study. Of these, 28 participants lacked sclerostin or metrnl test results, and three were excluded because their sclerostin (*n* = 2) or metrnl (*n* = 1) values were greater than the mean +2 standard deviation (SD) value. Accordingly, the results of 56 participants were included in this report.

This study’s processes followed those described in our previous studies (Mu et al., 2020; Mu et al., 2021). The IS portion of this study was conducted at IS sites in Liaoyang (12 January 2019) and Shenyang (26 January 2019, and 16 February 2019). The lengths of the swimming lanes at the three IS locations differed slightly, but all were between 25 and 30 m. All three winter swimming sessions had water temperatures below 5°C, a requirement for IS (Knechtle et al., 2021). On the day of IS, the participants were not required to fast, and they ate according to their daily dietary habits (they ate their usual amounts at the usual times); however, the participants were asked to eat a light diet (i.e., to avoid greasy and stimulating foods). All the participants commenced IS at 14:00.

The process of IS activities was performed in the following sequence: venous blood was drawn (30 min before IS) in an ambulance on the outdoor shore, and the participants performed warm-up preparations onshore. Then, they commenced IS (14:00) according to their own typical IS schedule (swimming time and distance were not mandated). Subsequently, the swimmers performed exercises on shore to recover their body temperature, and venous blood samples were again drawn in the ambulance (30 min after IS). The warm-up and recovery exercises (which usually included rope skipping, running, and push-ups) were performed according to each participant’s normal habits.

**FIGURE 1:**
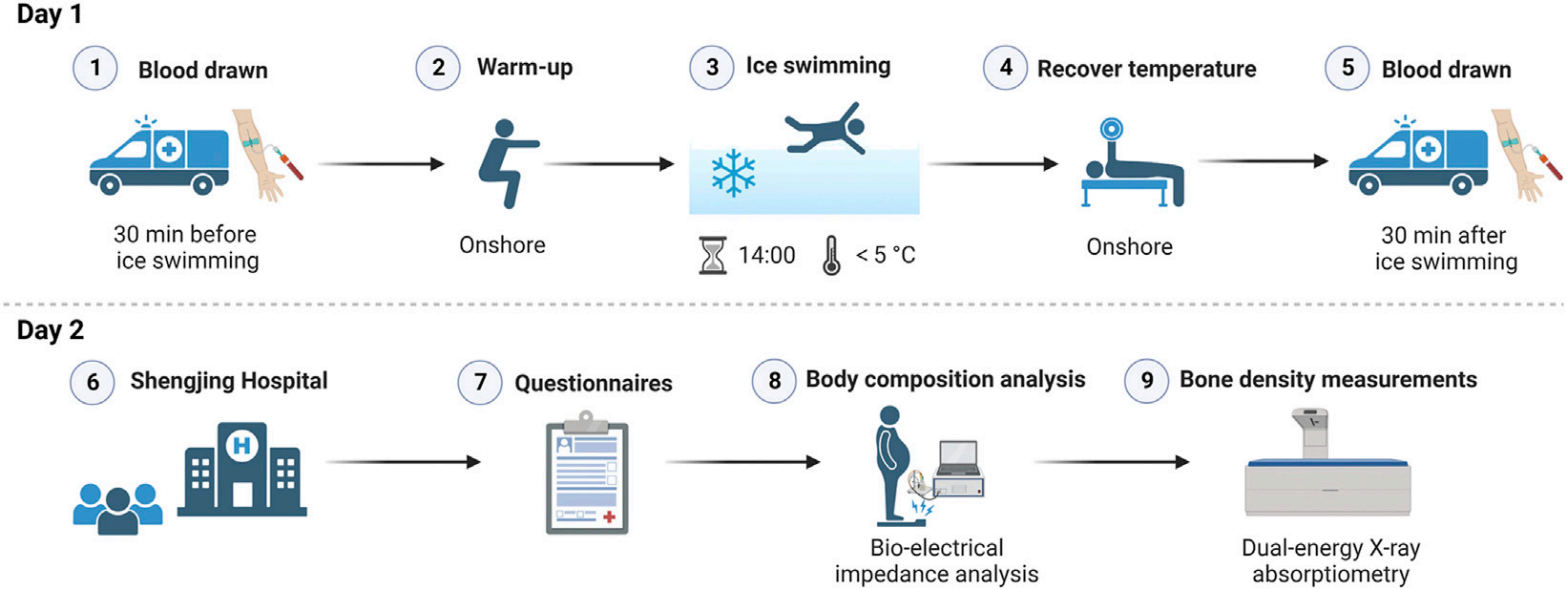
Schematic overview of the experimental procedures for the study. Created with BioRender.com.

The day following IS, all participants attended the Shengjing Hospital of China Medical University in the morning without fasting to complete questionnaires, body composition scans, and bone density measurements. The questionnaire recorded the participants’ respective IS distances from the previous day. The study protocol was approved by the Shengjing Hospital Ethics Committee of China Medical University and a schematic overview of the experimental protocol is presented in Figure 1.

### 2.2 Anthropometrics and bone density measurements

The participants were barefoot and wore only underclothes during the body composition measurements. Body composition was assessed using a multi-frequency impedance body composition analyzer (InBody 770; Biospace, Seoul, South Korea). The analyzer included the following body composition parameters: fat mass (FM), visceral fat area (VFA), fat-free mass (FFM), and skeletal muscle mass (SMM).

DXA scans of the lumbar spine (L1–L4) and proximal femur were acquired using the same method of dual-energy X-ray absorptiometry (Discovery-Wi S/N 88155; Hologic, Boston, MA, United States). To reduce the likelihood of error, the same experienced operator performed all scans using standardized procedures. The coefficient of variation for bone mineral density (BMD) was <1%.

### 2.3 Sclerostin and metrnl measurement

Blood samples were collected between 13:00 and 15:00 by trained nurses. Serum samples were obtained by centrifugation and stored at −80°C. Serum sclerostin levels were determined using commercial enzyme-linked immunosorbent assay (ELISA) kits (EK0968, Boster Biotech, Wuhan, China) according to the manufacturer’s instructions. The assay for sclerostin had a sensitivity of <10 pg/mL, and the intra-assay coefficient of variation was <8%. Serum metrnl levels were also measured using commercial ELISA kits (CSB-EL013718HU, Cusabio Biotech, Wuhan, China) according to the manufacturer’s instructions. The metrnl assay had a sensitivity of 0.039 ng/mL and an intra-assay coefficient of variation of <8%. The pre-IS scores represented the baseline circulating levels before IS, whereas Δ represented the circulating changes in response to IS.

### 2.4 Statistical analysis

The data were analyzed for normality using the Shapiro–Francia test. Continuous variable data with a normal distribution were presented as the mean and SD. By contrast, continuous variable data that were not normally distributed were presented as the median and the first to third quartiles. Before and after IS, differences in serum sclerostin and metrnl concentrations were evaluated using the Wilcoxon signed-rank matched paired test. Meanwhile, Spearman’s correlation coefficient analysis was conducted to determine the correlations between baseline sclerostin and baseline metrnl and between Δsclerostin and Δmetrnl. This study also used multivariate linear regression analysis models to examine the relationship between baseline sclerostin and baseline metrnl and between Δsclerostin and Δmetrnl after adjusting for age, gender, and body mass index (BMI) in Model I and after adjusting for age, gender, BMI, distance swum per session, FM, VFA, FFM, SMM, L1–L4 BMD, and femoral neck (FN) BMD in Model II. A two-sided *p*-value of <0.05 was deemed statistically significant. EmpowerStats software and the R (version 3.4.3) statistical package were used for the statistical analyses.

## 3 Results

### 3.1 Population characteristics

As shown in Table 1, this study included data from 56 participants, of whom 47 (83.9%) were male. The participants’ ages ranged from 45 to 84 years, with a mean (SD) age of 59.3 (8.3) years. As previously reported, there were no underweight (BMI <18.5 kg/m^2^) participants in the study (Mu et al., 2020). The IS distance for 85.7% of participants was <80 m. The average FM of the participants was 20.3 ± 5.8 kg, the median and inter-quartile range (IQR) of the participants’ VFA was 85.3 (72.4–104.7) cm^2^, the mean FFM of the participants was 53.4 ± 7.8 kg, the median and IQR of the participants’ SMM was 30.6 (26.5–32.8) kg, the average L1–L4 BMD of the participants was 1.0 ± 0.1 g/cm^2^, and the median and IQR of participants’ FN BMD was 0.8 (0.7–0.8) g/cm^2^.

**TABLE 1:** Baseline characteristics of participants (*N* = 56).

Characteristic	Statistics	Range
Males, *n*, %	47 (83.9%)	
Age, mean (SD), years	59.3 (8.3)	45.0–84.0
BMI, mean (SD), kg/m^2^	26.0 (2.7)	21.2–33.8
Ice swimming distance per session, n %, (meters)		
<80 m	48 (85.7%)	
≥80 m	8 (14.3%)	
FM, mean (SD), kg	20.3 (5.8)	10.1–35.8
VFA, median (Q1–Q3), cm^2^	85.3 (72.4–104.7)	47.2–178.9
FFM, mean (SD), kg	53.4 (7.8)	35.4–67.5
SMM, median (Q1–Q3), kg	30.6 (26.5–32.8)	8.3–38.4
L1–L4 BMD, mean (SD), g/cm^2^	1.0 (0.1)	0.7–1.2
FN BMD, median (Q1–Q3), g/cm^2^	0.8 (0.7–0.8)	0.6–1.3
Pre-IS sclerostin, median (Q1–Q3), pg/mL	114.0 (64.5–225.4)	4.5–540.1
Δsclerostin, median (Q1–Q3), pg/mL	–28.4 (–68.0–7.9)	–295.3–189.1
Pre-IS metrnl, median (Q1–Q3), ng/mL	0.2 (0.1–0.4)	0.0–0.9
Δmetrnl, median (Q1–Q3), ng/mL	0.0 (-0.1–0.1)	–0.5–0.5

Abbreviations: SD, standard deviation; Q1, first quartile; Q3, third quartile; BMI, body mass index; IS, ice swimming; FM, fat mass; VFA, visceral fat area; FFM, fat-free mass; SMM, skeletal muscle mass; L1–L4, lumbar spine 1–4; BMD, bone mineral density; FN, femoral neck; pre-IS, circulating levels before IS; Δ, circulating changes in response to IS.

### 3.2 Serum sclerostin and metrnl in response to ice swimming

**FIGURE 2:**
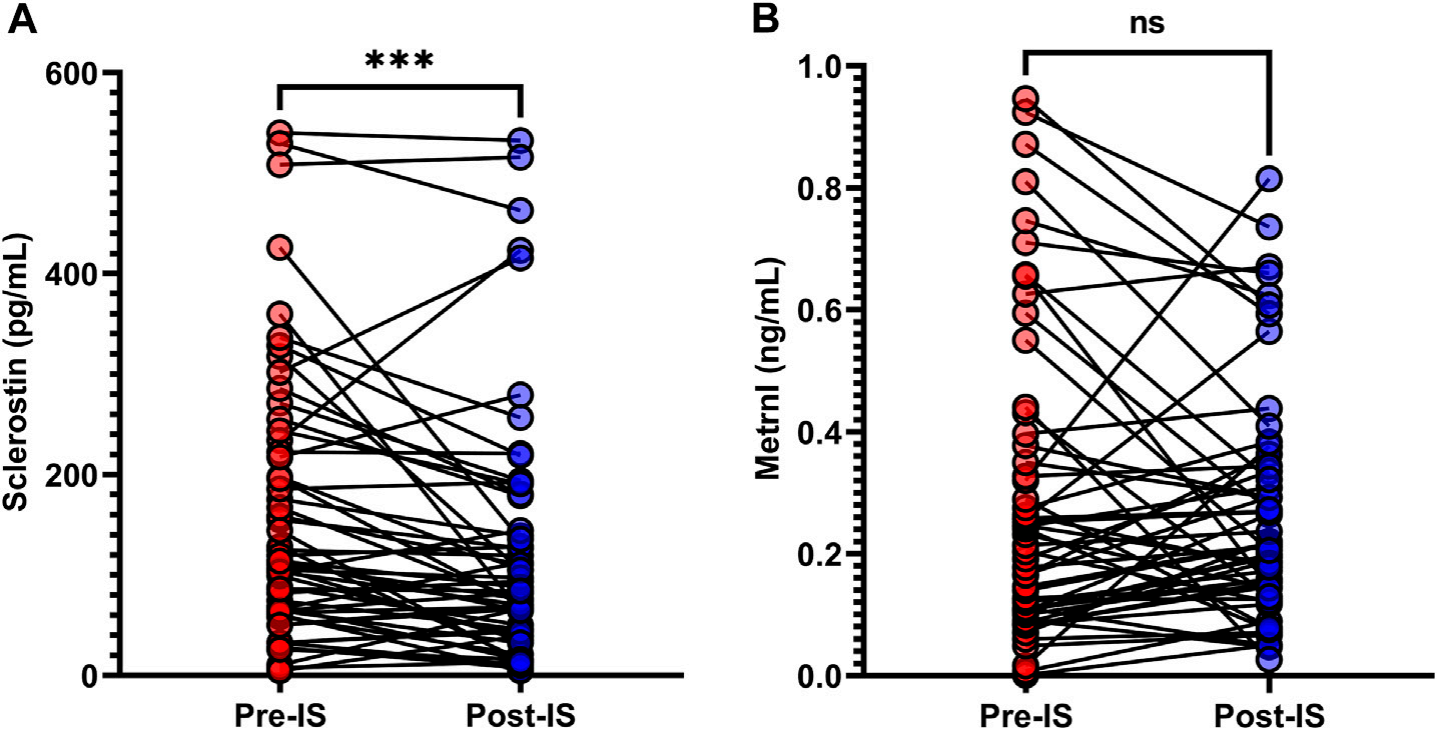
Variations in **(A)** serum sclerostin and **(B)** metrnl in response to ice swimming (IS). Wilcoxon’s signed-rank matched paired test was used to examine differences in serum sclerostin and metrnl concentrations before and after IS. ***p < 0.001.

As shown in Table 1 and Figure 2, the baseline sclerostin level of the total population was 114.0 (64.5–225.4) pg/mL, and the change in sclerostin following IS was −28.4 (−68.0–7.9) pg/mL. The baseline metrnl level was 0.2 (0.1–0.4) ng/mL, and the change in metrnl following IS was 0.0 (−0.1–0.1) ng/mL. There were significant decreases in sclerostin levels following IS (*p* < 0.001); however, serum metrnl levels did not change significantly (*p* = 0.861) after IS. In the male participants, significant decreases in sclerostin after IS were evident (Supplementary Figure S1), while the female participants did not exhibit a decrease in sclerostin after IS (Supplementary Figure S2). No significant change in metrnl was found after IS in both male and female participants (Supplementary Figures S1, S2).

### 3.3 Correlations between sclerostin/metrnl and anthropometric characteristics

The correlations between sclerostin/metrnl and anthropometric parameters are presented in Table 2, Supplementary Tables S1, S2. In all participants, only baseline sclerostin was positively correlated with L1–L4 BMD (Table 2). There were no associations between sclerostin/metrnl and anthropometric characteristics in the male population (Supplementary Table S1). However, in the female population, pre-IS sclerostin was positively correlated with FN BMD, and pre-IS metrnl was positively correlated with both FFM and SMM (Supplementary Table S2).

**TABLE 2:** Correlations between blood parameters and anthropometric characteristics.

Characteristic	BMI, kg/m^2^	FM, kg	VFA, cm^2^	FFM, kg	SMM, kg	L1–L4 BMD, g/cm^2^	FN BMD, g/cm^2^
Pre-IS sclerostin, pg/mL	–0.0109	–0.0153	–0.0273	–0.0061	0.0051	0.3180^*^	0.1952
Δsclerostin, pg/mL	–0.1454	–0.1511	–0.1223	0.0319	0.0359	–0.2104	–0.2205
Pre-IS metrnl, ng/mL	0.1230	0.0897	0.0978	0.1076	0.0986	0.2066	0.1145
Δmetrnl, ng/mL	–0.0930	–0.1423	–0.1393	–0.0203	–0.0633	–0.1959	–0.0394

Abbreviations: BMI, body mass index; IS, ice swimming; FM, fat mass; VFA, visceral fat area; FFM, fat-free mass; SMM, skeletal muscle mass; L1–L4, lumbar spine 1–4; BMD, bone mineral density; FN, femoral neck; pre-IS, circulating levels before IS; Δ, circulating changes in response to IS. *p < 0.05.

### 3.4 Correlations between sclerostin and metrnl

**FIGURE 3:**
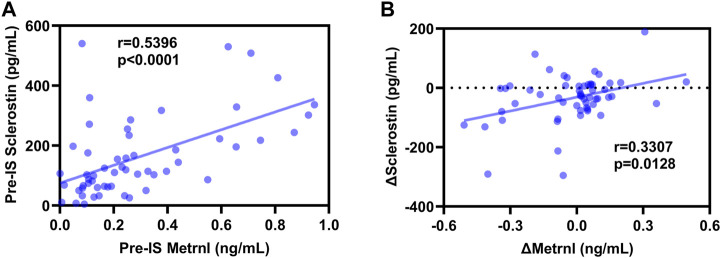
Correlations between sclerostin and metrnl. **(A)**: positive correlation between pre-ice swimming (IS) sclerostin and pre-IS metrnl; **(B)**: positive correlation between Δsclerostin and Δmetrnl.

As shown in Figure 3, Spearman’s correlation analyses revealed a positive association between baseline sclerostin and baseline metrnl (r = 0.5396, *p* < 0.0001), and Δsclerostin was positively associated with Δmetrnl (r = 0.3307, *p* = 0.0128) in all subjects. In the male participants, pre-IS sclerostin was positively correlated with pre-IS metrnl (r = 0.5823, *p* < 0.0001), while no significant associations were found between Δsclerostin and Δmetrnl (Supplementary Figure S3). In the female participants, there was no association between sclerostin and metrnl (Supplementary Figure S4).

### 3.5 Multivariate linear regression analysis of the associations between sclerostin and metrnl

In the multiple regression analyses, the unadjusted results revealed a positive correlation between the baseline levels of sclerostin and metrnl and a positive correlation between Δsclerostin and Δmetrnl in the total and male populations (Table 3 and Supplementary Table S3). The association between sclerostin and metrnl persisted after adjusting for covariates (Table 3 and Supplementary Table S3).

**TABLE 3:** Multivariate linear regression analysis of the relationship between baseline sclerostin–baseline metrnl and between Δsclerostin–Δmetrnl.

Independent variable	Dependent variable	Crude model		Model Ⅰ		Model Ⅱ	
		β (95%CI)	*p*-Value	β (95%CI)	*p*-Value	β (95%CI)	*p*-Value
Pre-IS metrnl	Pre-IS sclerostin	296.3 (178.8, 413.7)	<0.001	272.3 (154.9, 389.7)	<0.001	249.0 (129.3, 368.6)	<0.001
Δmetrnl	Δsclerostin	155.6 (52.4, 258.9)	0.005	158.7 (54.1, 263.4)	0.004	182.3 (68.7, 295.9)	0.003

Abbreviations/definitions: Crude, no adjustment; Model I, adjusted for age, gender, and body mass index (BMI); Model II, adjusted for age, gender, BMI, distance swum per session, fat mass, visceral fat area, fat-free mass, skeletal muscle mass, lumbar spine 1–4 bone mineral density (BMD), and femoral neck BMD; β, regression coefficient; CI, confidence interval.

## 4 Discussion

Both cold and exercise can affect the body’s adipose, bone, and muscle systems. Through investigations relating to typical traditional cold exercise (IS) (Tipton et al., 2017; Knechtle et al., 2021), we found that sclerostin, a key circulating osteokine, decreased significantly after IS. In contrast, no significant change in adipomyokine metrnl was observed before and after IS. In addition, we found that the baseline serum sclerostin level before IS was significantly and positively correlated with metrnl, and the decrease in sclerostin caused by IS was also significantly and positively correlated with metrnl fluctuations. To our knowledge, the present study is the first to report a correlation between circulating sclerostin and metrnl.

Our findings indicated a significant decrease in circulating sclerostin levels after cold exercise, which is in contrast with numerous studies that demonstrated significantly increased circulating sclerostin levels shortly after acute strenuous exercise. The acute effect of exercise on sclerostin is usually temporary, and the circulating sclerostin eventually returns to its baseline level after a finite time. For example, serum sclerostin levels in young men and women were significantly elevated 5 min after high-intensity exercise, but they returned to baseline levels 1 h later (Falk et al., 2016; Kouvelioti et al., 2018).

The reason why our findings are inconsistent with these acute exercise-induced sclerostin changes (excluding differences in age, gender, and the pattern, intensity, and duration of exercise) may be partly because our study included the effect of cold exposure stimulus in addition to that of exercise. However, as one of the few studies that have investigated the influence of cold on sclerostin concentration, Straburzyńska-Lupa et al. more recently reported that after whole-body cryostimulation (−110°C) in healthy young men, serum sclerostin levels increased slightly, although they did not reach statistical significance (Straburzynska-Lupa et al., 2021). Therefore, theoretically, IS, given the associated cold stimulation and acute exercise, could amplify the increase in circulating sclerostin levels.

However, we observed a significant decrease in sclerostin after IS. There is currently no plausible interpretation for this paradox. Nonetheless, the mechanisms underlying our finding that the suppression of sclerostin after IS may be related to the alterations in circulating hormone levels in cold exercise condition and/or increased renal elimination: 1) most evidence from both human and animal studies supports parathyroid hormone (PTH) is an important suppressor of sclerostin (Drake and Khosla, 2017), and we previously discovered that PTH increased significantly after IS (Mu et al., 2021), which is possibly related to sclerostin inhibition. However, when we analyzed the relationship between PTH rising and sclerostin falling in our study population, with no significant relationship identified (data not shown). Likewise, estrogen is recognized to has a suppressive effect on sclerostin levels (Drake and Khosla, 2017), and animal studies show an increase in 17-β estradiol levels in rats swimming in cold water (Bosiacki et al., 2021), which may be related to the decrease in sclerostin levels; and (2) although the mechanism(s) by which sclerostin is cleared from the circulation remains to be understood (Drake and Khosla, 2017), sclerostin is likely to be filtered across the glomerular membrane and is reabsorbed in the proximal tubule in physiological conditions (Cejka et al., 2014), hence decreased serum sclerostin levels may involved in the response of kidney function changes under the stress conditions of cold exercise. However, these hypotheses need to be confirmed.

The second finding of this study is that there was no significant change in metrnl after IS, which was inconsistent with our expectations, as both cold and exercise stimuli affect the secretion of metrnl (Rao et al., 2014). Most studies examining the correlation between exercise and metrnl levels indicate that exercise increases metrnl expression (Rao et al., 2014; Bae, 2018; Eaton et al., 2018; Alizadeh, 2022); however, some report that exercise does not affect blood metrnl (Bonfante et al., 2021).

Few studies have investigated the impact of environmental temperature during exercise on metrnl levels. Saghebjoo et al. reported that the metrnl level increased significantly after exercise at temperatures between 24°C and 25°C and in warm water (36.5°C–37.5°C) but decreased significantly in cold water (16.5°C–17.5°C), indicating that variations in environmental temperature during exercise affect metrnl levels (Saghebjoo et al., 2018). We observed no changes in metrnl after IS, which may have been related to our specific study population, cold exposure time, and exercise style.

However, we previously found a decrease in irisin levels after IS in the same subjects with this study (Mu et al., 2020), which suggested that the adipomyokines irisin and metrnl react differently to exercise in cold conditions. Furthermore, we previously found that irisin changes in response to cold exercise were correlated with body composition, particularly fat indicators (Mu et al., 2020). We analyzed the correlations between our body composition data, including BMI, adiposity indicators, muscle indicators, and BMD, with baseline metrnl and Δmetrnl, but found no positive correlations in the total and male populations (Table 2 and Supplementary Table S1). Nevertheless, there was a positive correlation between pre-IS metrnl and muscle indicators FFM and SMM in the female participants (Supplementary Table S2), suggesting that sex differences can affect the correlation between metrnl and body composition.

A particularly interesting finding in this study was the positive correlation between sclerostin and metrnl, both in terms of baseline levels and Δsclerostin and Δmetrnl. These correlations indicate the interconnectivity between biochemical signals in the body’s bone, muscle, and adipose systems. Previous research demonstrated a negative association between irisin and sclerostin in humans (Klangjareonchai et al., 2014). The underlying mechanism may be explained by irisin’s ability to promote the conversion of white adipose tissue to brown adipose tissue, thereby suppressing the expression of sclerostin (Klangjareonchai et al., 2014). Our research showed a positive correlation between metrnl and sclerostin, which may suggest that irisin and metrnl may impact sclerostin via different mechanisms. Notably, metrnl does not directly promote the browning of white adipose tissue, unlike irisin; rather, it stimulates several immune cell subtypes to enter adipose tissue and activate prothermogenic action (Rao et al., 2014).

Furthermore, owing to the features of this study’s design, we cannot yet establish a causal relationship between sclerostin and metrnl. Notably, despite the effect of irisin on sclerostin appears difficult to reconcile (Colaianni et al., 2015; Colaianni et al., 2017; Kim et al., 2019), previous studies have shown that irisin is a factor in regulating sclerostin, rather than the opposite (Colaianni et al., 2015; Colaianni et al., 2017; Kim et al., 2019), so similarly, metrnl may act on sclerostin. Nevertheless, future studies are needed to clarify the interplay between sclerostin and metrnl. In addition, two studies provided evidence that metrnl affects osteogenic differentiation (Gong et al., 2016; Huang et al., 2022). Although the conclusions of two experiments on whether metrnl promotes or inhibits osteogenic differentiation were inconsistent, both studies suggest that metrnl is predominantly expressed in osteoblasts (Gong et al., 2016; Huang et al., 2022). In contrast, osteocytes predominantly secrete sclerostin (although osteoblasts can also secrete small amounts of sclerostin) (Delgado-Calle and Bellido, 2022), suggesting a correlation between metrnl and sclerostin that may involve an associated regulation between osteocytes and osteoblasts. However, this hypothesis requires verification through further ground-up experiments.

This study has some limitations. First, there was a lack of control groups for both room-temperature swimming and cold non-swimming, which would have isolated the effects of cold or exercise on our findings. Second, despite having the largest number of subjects in the context of IS of any study, the sample size was small, with numerous males and few females among the enrolled subjects. Although it is possible that gender differences affected the results, the small number of female participants may have led to no significant changes in sclerostin after IS (Supplementary Figure S2), and no correlations between sclerostin and metrnl were observed (Supplementary Figure S4) in the female participants. This prevented us from incorporating the effect of other factors, such as menopause, into the results. Third, we did not test for immune cytokines correlated with metrnl, such as interleukin (IL)-4 and IL-13. Consequently, we were unable to detect any effect of these immune cytokines on the results. Fourth, we only examined sclerostin and metrnl levels once, 30 min after IS, which did not provide a complete picture of the changes in sclerostin and metrnl after a prolonged period (e.g., 24 h) following cold exercise. Fifth, we were unable to accurately detect the exercise intensity of each participant and observe the impact of exercise intensity on the research results. Lastly, no data on disease state (e.g., diabetes mellitus and cancer), the time interval between eating and IS, participants’ diets on the day of the test, or dietary habits were included in our study, so we could not exclude the effects of metabolic pathologies or dietary/macronutrient status on the outcomes.

## 5 Conclusion

The present study was conducted on a group of ice swimmers and showed that circulating sclerostin decreased significantly after IS, whereas circulating metrnl remained unchanged. According to the current data, the baseline sclerostin level and the change in circulating sclerostin in response to IS are correlated with the metrnl levels of ice swimmers. These results expand our understanding of the effects of cold exercise on osteokines and adipomyokines and provide new evidence on the interconnected network of the body’s bone, muscle, and adipose systems; this may lead to new insights into potential common therapeutic targets for diseases such as osteoporosis, sarcopenia, and obesity.

## Data Availability

The raw data supporting the conclusion of this article will be made available by the authors, without undue reservation.
