# Supplementary material for: Sclerostin decreases in regular swimmers after ice swimming and is associated with meteorin-like protein serum levels
Source: Front Physiol. 2023 Jun 26;14:1210457. doi: 10.3389/fphys.2023.1210457 (PMC10331292; doi:10.3389/fphys.2023.1210457)
Supplement: Supplementary file 1 [file DataSheet1.docx]

**
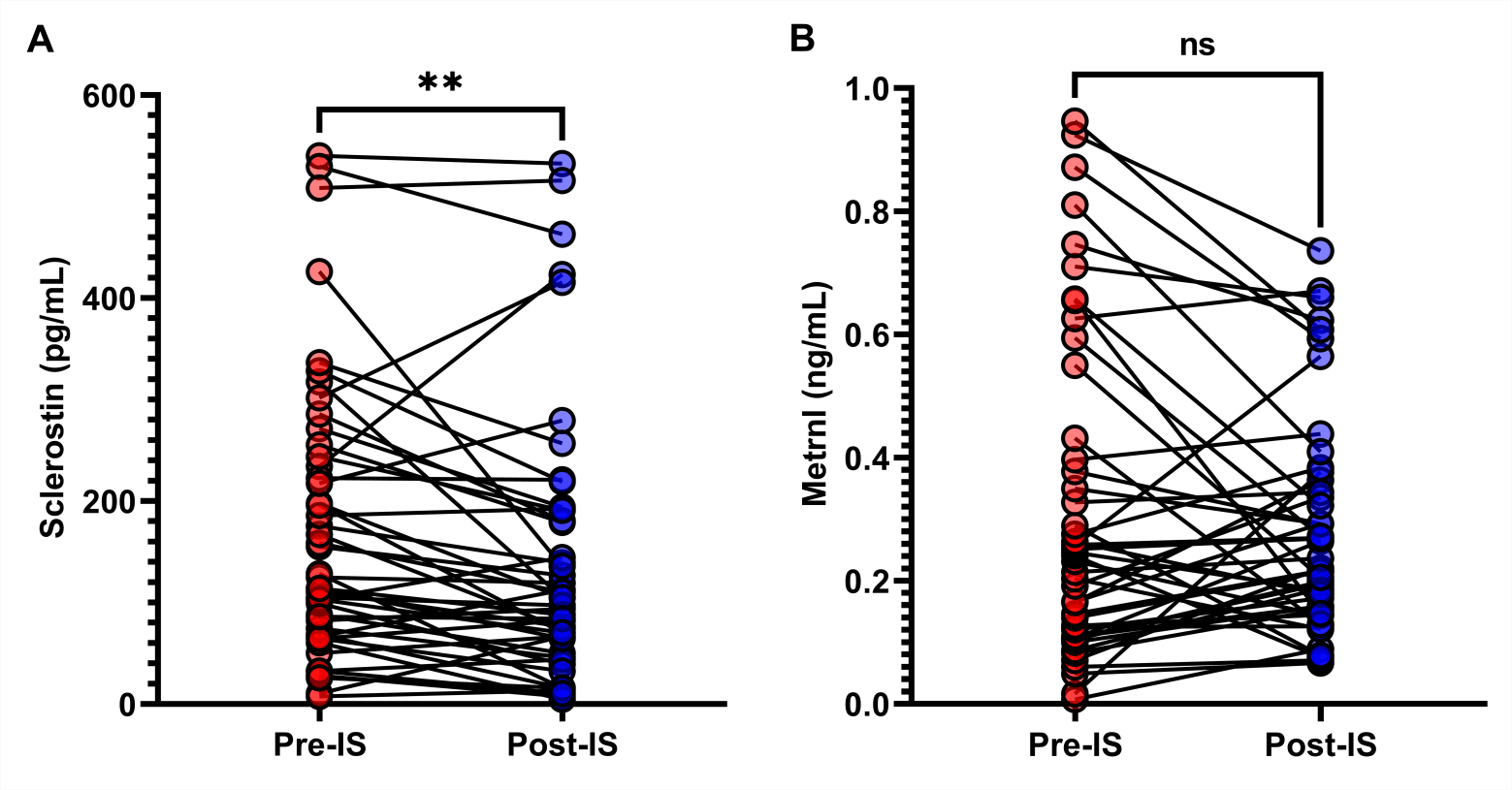
**

**Supplemental Figure 1. Variations in (A) serum sclerostin and (B) metrnl in response to ice swimming (IS) in male. Wilcoxon’s signed-rank matched paired test was used to examine differences in serum sclerostin and metrnl concentrations before and after IS. ***P* < 0.01.**

**
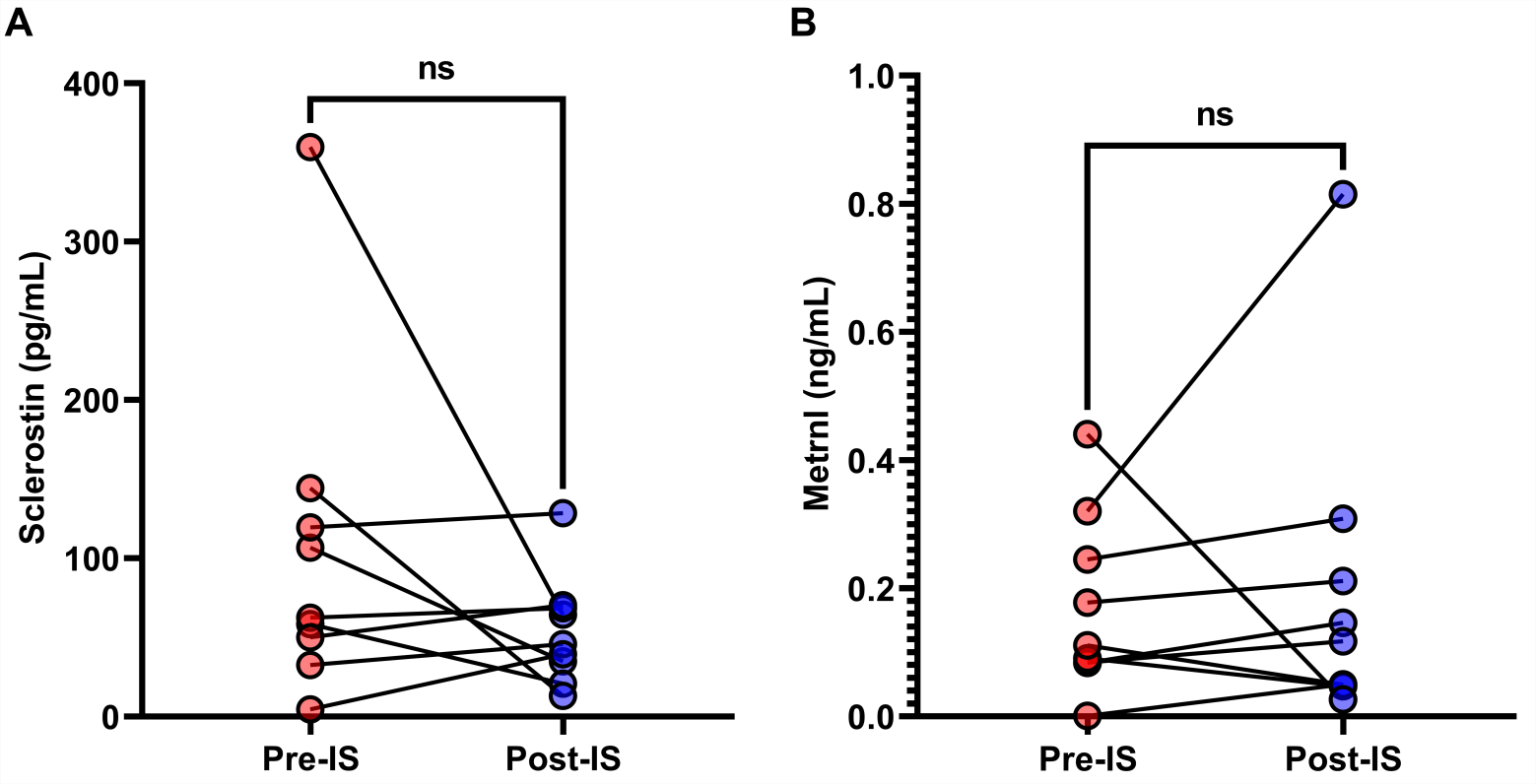
**

**Supplemental Figure 2. Variations in (A) serum sclerostin and (B) metrnl in response to ice swimming (IS) in female. Wilcoxon’s signed-rank matched paired test was used to examine differences in serum sclerostin and metrnl concentrations before and after IS.**

**Supplemental Table 1.** Correlations between blood parameters and anthropometric characteristics in male.

| Characteristic | BMI, kg/m^2^ | FM, kg | VFA, cm^2^ | FFM, kg | SMM, kg | L1–L4 BMD, g/cm^2^ | FN BMD, g/cm^2^ |
| --- | --- | --- | --- | --- | --- | --- | --- |
| Pre-IS sclerostin, pg/mL | –0.0340 | –0.0019 | –0.0353 | –0.2097 | –0.1827 | 0.2620 | 0.0572 |
| Δsclerostin, pg/mL | –0.1403 | –0.1240 | –0.0720 | 0.0709 | 0.0605 | –0.1398 | –0.1517 |
| Pre-IS metrnl, ng/mL | 0.1053 | 0.1241 | 0.1215 | –0.0715 | –0.0682 | 0.2313 | 0.1140 |
| Δmetrnl, ng/mL | –0.1305 | –0.1799 | –0.1565 | 0.0008 | –0.0477 | –0.1698 | 0.0239 |

Abbreviations: BMI: body mass index; IS: ice swimming; FM: fat mass; VFA: visceral fat area; FFM: fat-free mass; SMM: skeletal muscle mass; L1–L4: lumbar spine 1–4; BMD: bone mineral density; FN: femoral neck; pre-IS: circulating levels before IS; Δ: circulating changes in response to IS. * *P* < 0.05.

**Supplemental Table 2.** Correlations between blood parameters and anthropometric characteristics in female.

| Characteristic | BMI, kg/m^2^ | FM, kg | VFA, cm^2^ | FFM, kg | SMM, kg | L1–L4 BMD, g/cm^2^ | FN BMD, g/cm^2^ |
| --- | --- | --- | --- | --- | --- | --- | --- |
| Pre-IS sclerostin, pg/mL | 0.3833 | 0.4833 | 0.4833 | 0.2667 | 0.2667 | 0.6500 | 0.7500* |
| Δsclerostin, pg/mL | –0.2333 | –0.4000 | –0.4000 | –0.1500 | –0.1500 | –0.6333 | –0.6667 |
| Pre-IS metrnl, ng/mL | 0.1833 | 0.1167 | 0.1167 | 0.7000* | 0.7000* | –0.0833 | 0.0000 |
| Δmetrnl, ng/mL | 0.3167 | 0.1000 | 0.1000 | 0.1667 | 0.1667 | –0.2833 | –0.3667 |

Abbreviations: BMI: body mass index; IS: ice swimming; FM: fat mass; VFA: visceral fat area; FFM: fat-free mass; SMM: skeletal muscle mass; L1–L4: lumbar spine 1–4; BMD: bone mineral density; FN: femoral neck; pre-IS: circulating levels before IS; Δ: circulating changes in response to IS. * *P* < 0.05.

**
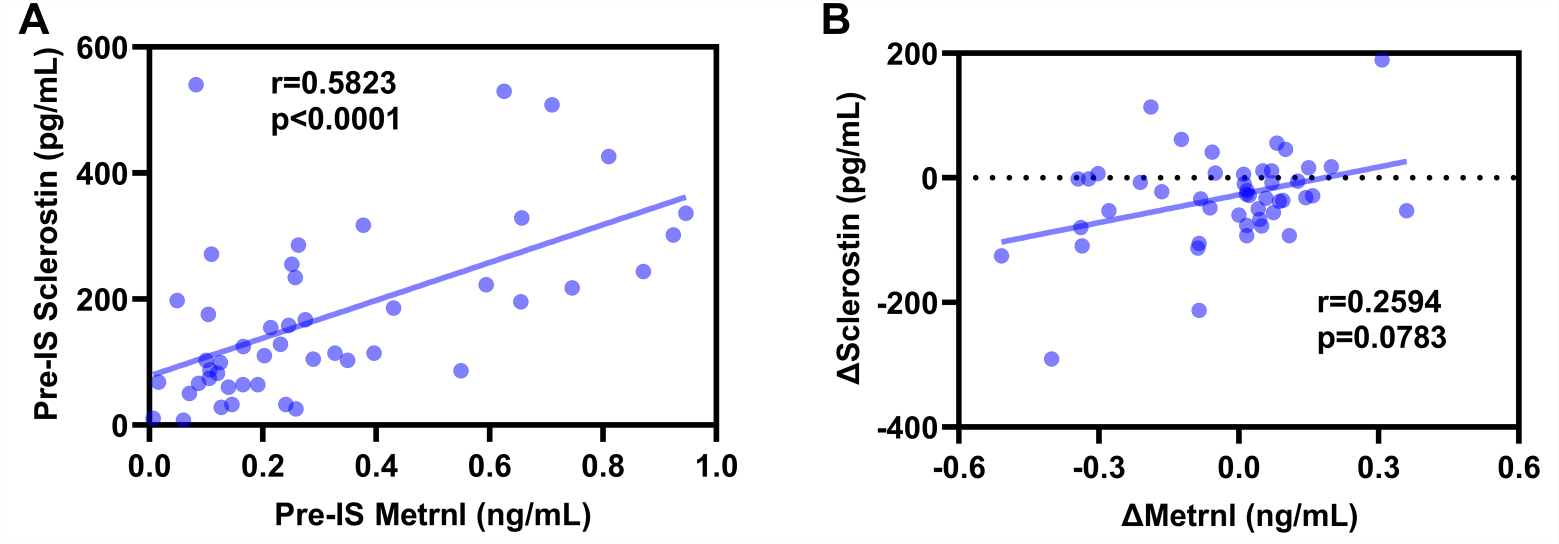
**

**Supplemental Figure 3. Correlations between sclerostin and metrnl in male. (A): positive correlation between pre-ice swimming (IS) sclerostin and pre-IS metrnl; (B): no significant correlation between Δsclerostin and Δmetrnl.**

**
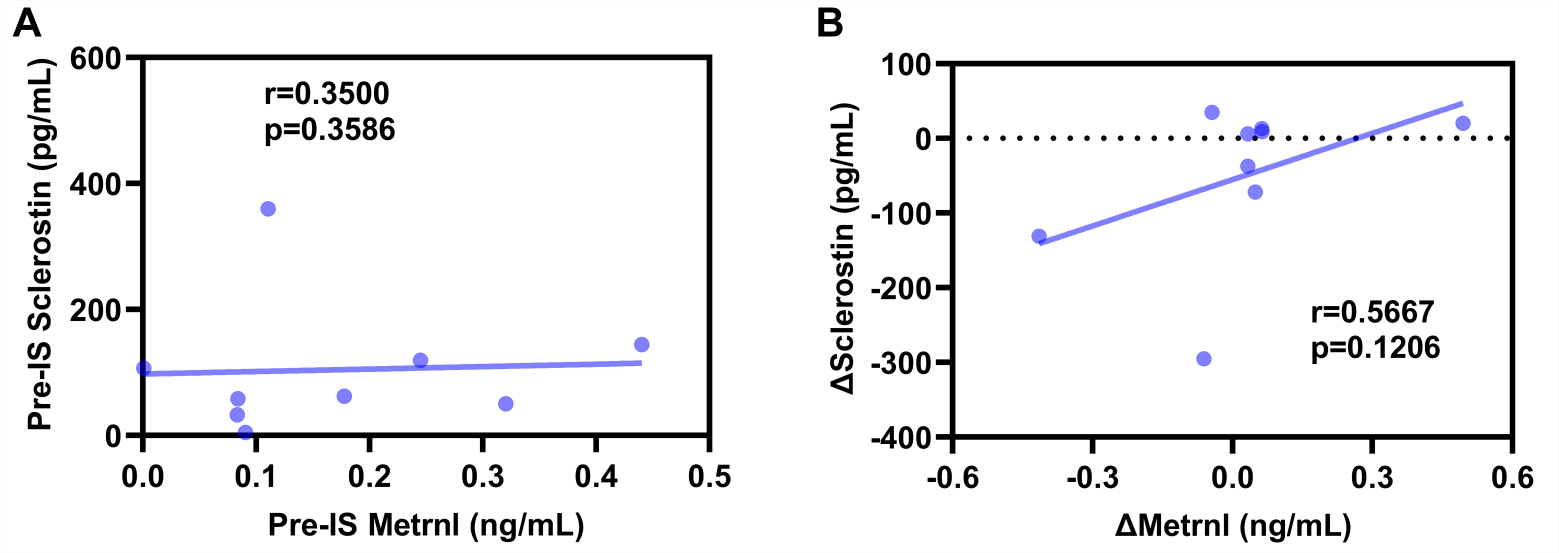
**

**Supplemental Figure 4. Correlations between sclerostin and metrnl in female. (A):no significant correlation between pre-ice swimming (IS) sclerostin and pre-IS metrnl; (B): no significant correlation between Δsclerostin and Δmetrnl.**

**Supplemental Table 3.** Multivariate linear regression analysis of the relationship between baseline sclerostin–baseline metrnl and between Δsclerostin–Δmetrnl in male.

| Independent variable | Dependent variable | Crude Model | |  | Model Ⅰ | |  | Model Ⅱ | |
| --- | --- | --- | --- | --- | --- | --- | --- | --- | --- |
|  |  | β (95%CI) | *P*-value |  | β (95%CI) | *P*-value |  | β (95%CI) | *P*-value |
| Pre-IS metrnl | Pre-IS sclerostin | 300.1 (176.2, 424.0) | <0.001 |  | 282.6 (159.8, 405.5) | <0.001 |  | 256.0 (127.5, 384.5) | <0.001 |
| Δmetrnl | Δsclerostin | 149.0 (38.5, 259.6) | 0.011 |  | 146.9 (35.1, 258.8) | 0.014 |  | 162.4 (39.6, 285.1) | 0.014 |

Abbreviations/definitions: Crude: no adjustment; Model I: adjusted for age, gender, and body mass index (BMI); Model II: adjusted for age, gender, BMI, distance swum per session, fat mass, visceral fat area, fat-free mass, skeletal muscle mass, lumbar spine 1–4 bone mineral density (BMD), and femoral neck BMD; β: regression coefficient; CI: confidence interval.
